# Rab18 binds to classical swine fever virus NS5A and mediates viral replication and assembly in swine umbilical vein endothelial cells

**DOI:** 10.1080/21505594.2020.1767356

**Published:** 2020-05-18

**Authors:** Liang Zhang, Di Zhao, Mingxing Jin, Mengzhao Song, Shanchuan Liu, Kangkang Guo, Yanming Zhang

**Affiliations:** College of Veterinary Medicine, Northwest A&F University, Yangling, Shaanxi, China

**Keywords:** Classical swine fever virus, Rab18, NS5A, interaction, replication, assembly

## Abstract

Classical swine fever virus (CSFV), a positive-sense RNA virus, hijacks cell host proteins for its own replication. Rab18, a small Rab GTPase, regulates intracellular membrane-trafficking events between various compartments in cells and is involved in the life cycle of multiple viruses. However, the effect of Rab18 on the production of CSFV remains uncertain. In this study, we showed that knockdown of Rab18 by lentiviruses inhibited CSFV production, while overexpression of Rab18 by lentiviruses enhanced CSFV production. Subsequent experiments revealed that the negative-mutant Rab18-S22 N inhibited CSFV infection, while the positive-mutant Rab18-Q67 L enhanced CSFV infection. Furthermore, we showed that CSFV RNA replication and virion assembly, measured by real-time fluorescence quantitative PCR (RT-qPCR), indirect immunofluorescence assay (IFA), and confocal microscopy, were reduced in cells lacking Rab18 expression. In addition, co-immunoprecipitation, GST-pulldown, and confocal microscopy assays revealed that Rab18 bound to the viral protein NS5A. Further, NS5A was shown to be redistributed in Rab18 knockdown cells. Taken together, these findings demonstrate Rab18 as a novel host factor required for CSFV RNA replication and particle assembly by interaction with the viral protein NS5A.

## Introduction

Classical swine fever (CSF), a highly infectious disease caused by classical swine fever virus (CSFV), is one of the diseases notifiable to the Office International Des Epizooties (OIE). CSFV, a member of the *Flaviviridae* family, is an enveloped virus with a 12.3-kb plus-strand RNA genome, carrying one large open reading frame (ORF) flanked by 5′ and 3′ untranslated regions (UTR) [1–3]. The ORF encodes a 3898 amino acid polyprotein precursor, which is cleaved by viral proteases and host proteases into 12 different proteins, including four structural proteins (Core, E^rns^, E1, and E2) and eight non-structural proteins (N^pro^, p7, NS2, NS3, NS4A, NS4B, NS5A, and NS5B) [4,5].

The CSFV NS5A protein is a 55-kDa protein containing 497 amino acids and primarily localized in the endoplasmic reticulum (ER) of the host cell [6]. Similar to hepatitis C virus (HCV) and bovine viral diarrhea virus (BVDV), other members of the *Flaviviridae* family, the CSFV NS5A protein is also a zinc metalloprotein [7–10]. Together with other non-structural proteins (i.e., NS3, NS4A, NS4B, and NS5B), it forms an RNA replicase complex, which is involved in viral RNA replication [11]. The conserved sequence C2717-C2740-C2742-C2767 of NS5A is essential for viral replication [10]. Additionally, another conserved sequence in the C-terminal region (amino acids 478–487) of NS5A protein is required for virion assembly [12]. Although the definitive mechanism of the NS5A protein in the CSFV life cycle and pathogenesis remains unknown, its mechanism in modulating the host cell environment has been confirmed. Studies proved that the interactions between host cell proteins and NS5A were essential for infectious virus production. Previous studies showed that the NS5A protein interacts with the 3ʹ-UTR, 5ʹ-UTR, and NS5B protein [13,14]. Moreover, the host proteins ANXA2, HSP70, and Rab1A (identified as NS5A binding proteins) enhance CSFV production [15–17].

Rab GTPases are a superfamily of small GTPases, which contains approximately 70 Rab proteins in eukaryotic cells [18]. Rab proteins are known as regulators of vesicular transport in the cycling between a GDP-bound inactive form and GTP-bound active form [19,20]. Rab18, belonging to the Rab GTPases family, is located in the endoplasmic reticulum (ER), Golgi apparatus and lipid droplets (LDs), and plays a central role in vesicular transport from the Golgi to the ER [21–23]. In Warburg Micro Syndrome, Rab18 is a critical regulator of neuronal migration and morphogenesis [24]. In addition, Rab18 promotes the growth and chemoresistance of gastric cancer by regulating mitochondrial function [25]. Previous studies showed that Rab18 was involved in the proliferation of numerous viruses. Rab18 is essential for HCV assembly through trafficking of the viral protein core and NS5A to LDs [26,27]. Rab18 is also an important host factor for BK polyomavirus (BKPyV) and dengue virus (DENV) infection [28,29]. Recently, Rab1, Rab5, Rab7, and Rab11 were identified as essential host factors for CSFV invasion and replication [17,30]. However, the function of Rab18 in the CSFV life cycle has not been illustrated.

In this study, we demonstrated the positive role of Rab18 in CSFV infection using Rab18 knockdown and Rab18 overexpression cell lines as well as Rab18(Q67L)- and Rab18(S22N)-transfected cells. Our results demonstrated Rab18 as an essential host factor for CSFV production in multiple life cycles, including viral replication and particle assembly, through binding of the viral protein NS5A. Further, we characterized Rab18 as a novel factor in the host-virus protein molecular interaction network of CSFV replication, which may represent a potential antiviral strategy for anti-CSFV treatment.

## Materials and methods

### Cells and viruses

The swine umbilical vein endothelial cell lines (SUVECs) conserved in our laboratory were cultured in Medium 199 (Gibco, Cat 11150059) with 10% fetal bovine serum (FBS) (Gibco, Cat 10099141 C), 50 μg/mL heparin (Sigma-Aldrich, Cat 375095), and penicillin-streptomycin solution (Sigma-Aldrich, Cat V900929) [31]. Human embryonic kidney (HEK293 T) cells (ATCC: CRL-11268) were maintained in high glucose DMEM (Gibco, 11965092) with 10% FBS and penicillin-streptomycin solution. The CSFV Shimen strain (GenBank: AF092448) was obtained from the China Institute of Veterinary Drug Control (Beijing, China) and propagated in PK-15 cells. All these cells were cultured in an incubator at 37°C under 5% CO_2_.

### Plasmid construction

Total cellular RNA was isolated from SUVECs using RNAiso Plus (Takara Bio, Cat 9108), and cDNA was synthesized using the PrimeScript™ 1st Strand cDNA Synthesis Kit (Takara Bio, Cat 6110A). The swine Rab18 gene (Gene ID: 100620478) was amplified from cDNA by polymerase chain reaction (PCR) and cloned into the vectors pcDNA-Flag-Red, pGEX-6p-1 (GE Healthcare, 28–9546-48), and pCDH-CMV-MCS-EF1 (SBI, Cat CD513B-1) to generate pcDNA-Flag-Red-Rab18, pGEX-GST-Rab18, and pCDH-CMV-Rab18, respectively. The GDP-bound inactive form Rab18-S22N and the GTP-bound active form Rab18-Q67L were generated by the single-primer mutagenesis method and cloned into the vector pcDNA-Flag-Red to obtain pcDNA-Flag-Red-Rab18 (S22N) and pcDNA-Flag-Red-Rab18 (Q67 L), respectively. CSFV NS2 and NS5A genes were separately inserted into the pcDNA3.1 (+) vector to generate pcDNA-NS2-Myc and pcDNA-NS5A-Myc, respectively. The NS5A gene was also inserted into pEGFP-N1 to obtain pEGFP-NS5A. Three pairs of shRNAs targeting the swine Rab18 gene and a negative control NTshRNA were predicted (http://rnaidesigner.thermofisher.com/) and designed. After annealing, the fragments were cloned into pCDH-U6-Green Puro (SBI, Cat SI505A-1) to create shRab18-1, shRab18-2, shRab18-3, and NTshRNA lentivectors, respectively. All primers used in this study are listed in Table 1.

**Table 1.** Primers used in this study.

Primers	Sequence (5′-3′)	Application
NTshRNA-F NTshRNA-R	GATCCGCTTAAACGCATAGTAGGACTCAAGAGAGTCCTACTATGCGTTTAAGCTTTTTG AATTCAAAAAGCTTAAACGCATAGTAGGACTCTCTTGAGTCCTACTATGCGTTTAAGCG	RNAi
shRab18-1-F shRab18-1-R	GATCCGGATGGAAATAAGGCTAAACTCAAGAGAGTTTAGCCTTATTTCCATCCTTTTTG AATTCAAAAAGGATGGAAATAAGGCTAAACTCTCTTGAGTTTAGCCTTATTTCCATCCG	RNAi
shRab18-2-F shRab18-2-R	GATCCGGTGCACAGGGTGTTATATTACAAGAGTAATATAACACCCTGTGCACCTTTTTG AATTCAAAAAGGTGCACAGGGTGTTATATTACTCTTGTAATATAACACCCTGTGCACCG	RNAi
shRab18-3-F shRab18-3-R	GATCCGGCCTGAAATTTGCACGAAAGCAAGAGCTTTCGTGCAAATTTCAGGCCTTTTTG AATTCAAAAAGGCCTGAAATTTGCACGAAAGCTCTTGCTTTCGTGCAAATTTCAGGCCG	RNAi
Rab18-mut-F Rab18-mut-R	TCGTGAAGTTGACAGAAATGAACGCCCGAATTATGAACAAAACCATTCCATG CATGGAATGGTTTTGTTCATAATTCGGGCGTTCATTTCTGTCAACTTCACGA	Cloning
Rab18-F Rab18-R	ATGGACGAGGACGTGCTGACTAC TGAACCTCAAGAGCAGGCTGGAC	RT-qPCR
β-actin-F β-actin-R	CAAGGACCTCTACGCCAACAC TGGAGGCGCGATGATCTT	RT-qPCR
CSFV-F CSFV-R	GAGAAGGACAGCAGAACTAAGC TTACCGCCCATGCCAATAGG	RT-qPCR
Rab18-WT-F Rab18-WT-R	CGGGATCCGCCACCATGGACGAGGACGTGCT CGGAATTCTTATAACACAGAACAATAACCACC	Cloning
Rab18-Q67L-F Rab18-Q67L-R	GGGATACTGCTGGTCTAGAAAGGTTCAGAAC CTTCTGAACCTTTCTAGACCAGCAGTATCCC	Cloning
Rab18-S22N-F Rab18-S22N-R	AGTGGCGTGGGCAAGAACAGCCTGCTCTTG TCAAGAGCAGGCTGTTCTTGCCCACGCCAC	Cloning
GFP-NS5A-F GFP-NS5A-R	CCCAAGCTTATGTCAAGTAATTACATTCTAGAGCTCC TCCCCGCGGCAGTTTCATAGAATACACTTTTGC	Cloning
NS5A-Myc-F NS5A-Myc-R	CGGGATCCGCCACCATGTCAAGTAATTACATTCTAGAGCTCC CGGAATTCTTACAGATCCTCTTCAGAGATGAGTTTCTGCTCCAGTTTCATAGAATACACTTTTGC	Cloning
GST-Rab18-F GST-Rab18-R	CGGGATCCGCCACCATGGACGAGGACGTGCT CGGAATTCTTATAACACAGAACAATAACCACC	Cloning

### Lentivirus production

Rab18 lentiviruses (overexpression and knockdown) were produced as previously described [16]. Briefly, overexpression or knockdown plasmids with three other plasmids (pGag/Pol, pRev, pVSVG) were co-transfected into HEK293 T cells using Lipofectamine 3000 Transfection Reagent (Thermo Fisher Scientific, Cat L3000015). After 16 h, the medium was replaced with advanced DMEM containing 2% FBS, 0.01 mmol/L cholesterol (Sigma-Aldrich, Cat C8667), 0.01 mmol/L L-α-phosphatidylcholine (Sigma-Aldrich, Cat P443), 1:1000 diluted Chemically Defined Lipid (Invitrogen, Cat 11905031), and 4 mmol/L L-glutamine (Sigma-Aldrich, Cat G7513). After 48 h, supernatants containing the lentivirus were collected. Lentiviral titers were determined by tissue culture infectious dose (TCID50) in HEK293 T cells. The lentiviruses (MOI = 1) were applied to infect SUVECs. After a 12-h infection, SUVECs were cultured in fresh medium with puromycin (5 mg/mL) (Sigma-Aldrich, Cat P8833) to select stable cell lines. The empty vector CMV and random sequence vector (NTshRNA) were treated equally as controls.

### Cell viability assay

The viability of cells was measured using Cell Counting Kit-8 (CCK-8) (Dojindo, Cat CK04) according to the manufacturer’s instructions. Briefly, a series of SUVECs were seeded into 96-well microplates and cultured for 48 h. Subsequently, 10 µL of the cell viability reagent was directly added to each well and incubated for 2 h at 37°C. The absorbance values were recorded at 450 nm using the SpectraMax M5 Microplate Reader (Molecular Devices, San Francisco, CA, USA).

### RNA extraction and quantitative reverse transcription PCR (RT‑qPCR)

RT-qPCR was conducted to detect the expression of Rab18 mRNA and CSFV RNA using specific primers listed in Table 1. Total cellular RNA was extracted using RNAiso Plus (Takara Bio, Cat 9108). Virus RNA in cell cultures was extracted using the TIANamp Virus RNA Kit (TIANGEN Biotech, Cat DP315-R). The RNA was quantified using the NanoDrop 1000 spectrophotometer (Nano Drop Technologies, Wilmington, DE, USA). cDNA was synthesized using the Evo M-MLV RT for PCR Kit (Accurate Biotechnology, Cat AG11604). RNA expression was normalized to the housekeeping gene β-actin, estimated using the TB Green™ Premix Ex Taq™ II (Takara Bio, Cat RR820A) according to the manufacturer’s protocol, and tested using the Applied Biosystems 7500 Real-Time PCR system (Life Technologies, Carlsbad, CA, USA). Data were analyzed according to the comparative threshold (Ct) method.

### Virus titration by indirect immunofluorescence assay (IFA)

IFA and viral titration were performed as previously described [32]. Cells seeded in collagen-coated 96-well plates were infected with virus. After 72 h, the cells were fixed with 4% paraformaldehyde for 20 min. Following three washes with PBS, the fixed cells were penetrated with 0.5% Triton X-100 for 10 min. After three washes with PBS, the cells were treated with 3% BSA for 2 h. Subsequently, the cells were incubated with mouse anti-E2 antibody (1:200) (Ab-mart) at 4°C for 12 h. After three washes with PBS, the cells were incubated with FITC-conjugated goat anti-mouse antibody (1:200) (Sigma-Aldrich, Cat IP124 F) for 1 h at room temperature. The fluorescence-positive wells were observed under a fluorescence inversion microscope (Nikon, Tokyo, Japan). The viral titers were determined as (TCID50) /mL by the method of Reed and Muench.

### Confocal microscopy

The cells were seeded on glass coverslips in 35-mm cell culture dishes and cultured overnight. Thereafter, the SUVECs were co-transfected with pEGFP-N1 and pcDNA-Flag-Red, pEGFP-NS5A and pcDNA-Flag-Red-Rab18, pEGFP-NS5A and pcDNA-Flag-Red-Rab18 (S22N), and pEGFP-NS5A and pcDNA-Flag-Red-Rab18 (Q67L). After 24 h, the cells were washed three times with cold PBS and fixed with 4% paraformaldehyde for 20 min at room temperature. Subsequently, the cells were incubated with DAPI at 37°C for 10 min and washed with cold PBS. Finally, images were captured by laser scanning confocal microscopy (LSM510 META, Zeiss, Germany).

### Co-immunoprecipitation (Co-IP) assays

SUVECs cultured in 6-well plates were co-transfected with the indicated plasmids (pcDNA-Flag-Red-Rab18, pcDNA-Flag-Red-Rab18 (S22N), and pcDNA-Flag-Red-Rab18 (Q67L); pcDNA-NS2-Myc and pcDNA-NS5A-Myc) using the Lipofectamine 3000 Transfection Reagent. After 48 h, the cells were harvested and lysed in western blot or IP lysis buffer containing protease inhibitor cocktail (Sigma-Aldrich, Cat P8340). After incubating on ice for 30 min, the cell lysates were centrifuged at 13000 g for 30 min. Approximately 25% of the supernatant was subjected to input assays, and the remaining was used for the Co-IP assay with an anti-c-Myc agarose affinity gel (Sigma-Aldrich, Cat IP0020) according to the manufacturer’s instructions. Briefly, 50 µL of the agarose affinity gel was centrifuged for 30 s at 4°C to remove glycerol and washed with cold TBS. The cell lysate was added to the equilibrated resin and rocked gently on a rotating platform at 4°C overnight. The resin was washed with cold TBS, and the protein samples were evaluated by western blot.

### GST-pulldown assays

The plasmids pGEX-6p-1 and pGEX-GST-Rab18 were transformed into BL21 competent cells (Invitrogen, Carlsbad, CA, United States) to obtain GST and GST-Rab18 proteins, respectively. The plasmid pcDNA-NS5A-Myc was transfected into HEK-293 T cells to obtain the NS5A-Myc protein. The Pierce GST Protein Interaction Pull-Down Kit (Thermo Fisher Scientific, Cat 21516) was used according to the manufacturer’s instructions. Briefly, BL21 cells containing GST or GST-Rab18 proteins were treated with pull-down lysis buffer and immobilized on equilibrated glutathione agarose resin at 4°C for 2 h. The resin was washed with wash solution, and HEK293 T lysates containing NS5A-Myc protein were added, followed by incubation at 4°C for 12 h. After washing with wash solution, the resin was eluted with glutathione elution buffer. The protein samples were evaluated by western blot.

### Western blot

Protein samples were separated by 10% SDS-PAGE, followed by transferring to polyvinylidene difluoride (PVDF) membranes (Merck Millipore, Cat ISEQ00010). After blocking with 5% milk, the membranes were incubated with the primary antibodies mouse anti-Flag antibody (1:3000) (Proteintech, Cat 66008-3-Ig), mouse anti-Myc antibody (1:3000) (Proteintech, Cat 60003-2-Ig), mouse anti-Rab18 antibody (1:500) (Santa Cruz Biotechnology, Cat sc-393168), mouse anti-GST antibody (1:2000) (Abcam, Cat ab19256), or rabbit anti-actin antibody (1:5000) (Abcam, Cat ab8227) at 4°C for 12 h. Subsequently, the membranes were incubated with horseradish peroxidase (HRP)-conjugated goat anti-mouse IgG (Proteintech, Cat SA00001-1) secondary antibody (1:5000) for 2 h. Finally, the signal was detected using an enhanced chemiluminescence (ECL) western blot analysis system. β-actin served as an internal control protein.

### Virus binding and entry assay

Cells were infected with CSFV at 4°C to achieve viral binding but impair viral entry. The excess virus was then washed away using PBS. CSFV RNA collected at this point was considered to represent CSFV bound to the cell surface. To promote virus entry, cells were treated as described above for virus binding and then incubated at 37°C to allow CSFV entry. Viral RNA collected at this point was considered to represent CSFV that had entered the cells. RT-qPCR was subsequently performed with intracellular CSFV genomic RNA.

### Statistical analysis

All experiments were performed at least three times. All data were expressed as mean ± standard deviation (SD). The data were analyzed by *t*-test with GraphPad Prism 6 software (GraphPad Software, Inc., La Jolla, CA, USA). *P* < 0.05 was considered significant.

## Results

### Knockdown of Rab18 suppresses CSFV production

**Figure 1.**
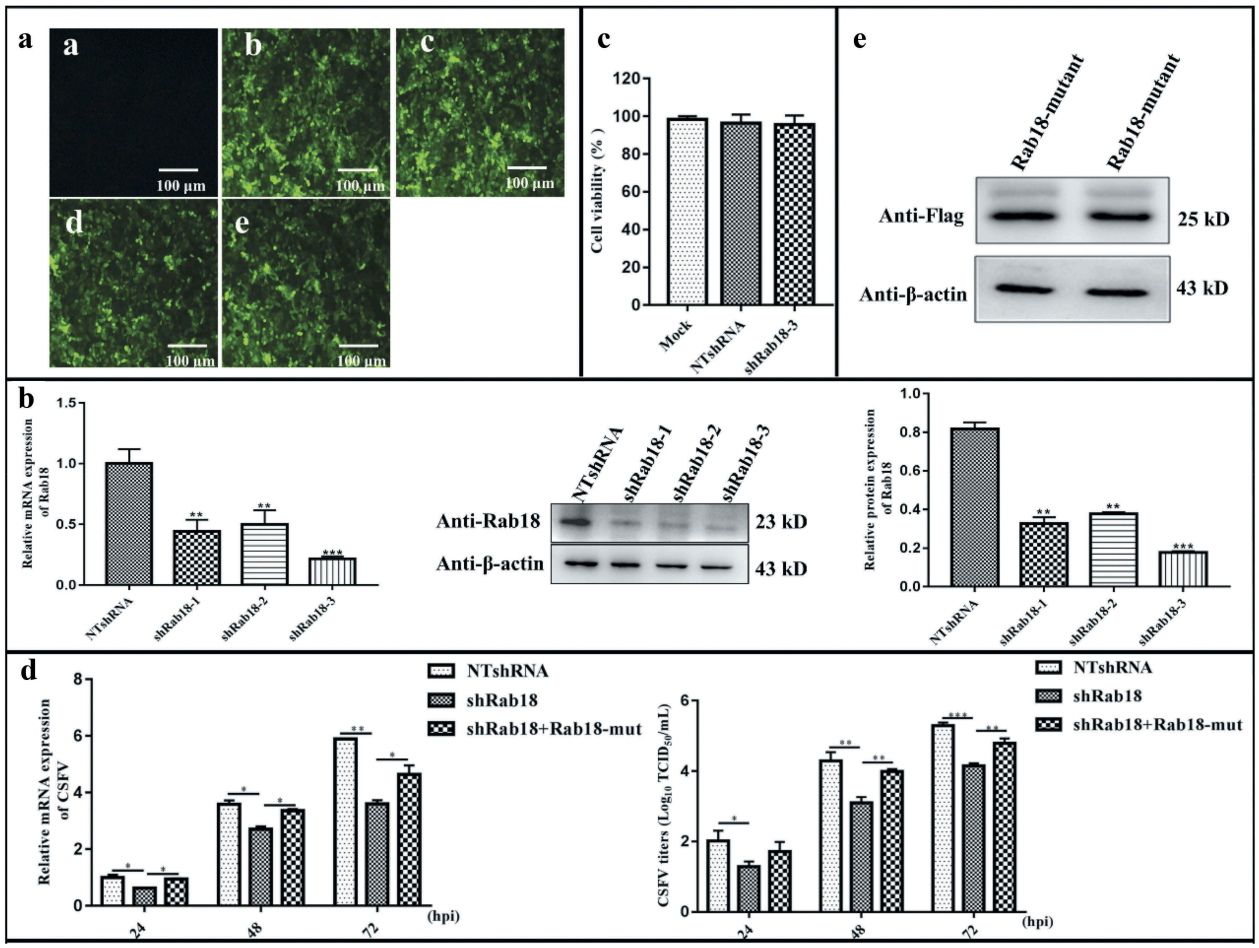
Knockdown of Rab18 inhibits CSFV production. (a–c) Knockdown of Rab18 in SUVECs by lentivirus-mediated shRNA interference. (a) Confirmation of NTshRNA, shRab18-1, shRab18-2, and shRab18-3 cell lines by detection of the enhanced green fluorescent protein (EGFP) reporter. (**a**) Mock-transfected SUVECs. (b) SUVECs transfected with lentiviruses expressing NTshRNA. SUVECs transfected with lentiviruses expressing (c) shRab18-1, (d) shRab18-2, or (e) shRab18-3. Scale bars, 100 μm. (b) qRT-PCR and western blot analyze Rab18 gene and protein expression levels in Rab18 knockdown cell lines. β-actin served as an internal control. (c) Cell viability of shRab18-3 cell lines. (d) qRT-PCR analyzes CSFV RNA level and TCID50L assay to detect CSFV viral titers in the supernatants of NTshRNA cell lines, shRab18-3 cell lines, and Rab18-mutant-transfected cells. (e) Western blot analyzes Rab18-mutant in transfected cells using an anti-Flag antibody. β-actin served as an internal control.

To elucidate the role of endogenous Rab18 in CSFV infection, three different short hairpin RNA (shRNA) sequences (shRab18-1, shRab18-2, shRab18-3) against *Rab18* and a non-targeting shRNA (NTshRNA) were designed and separately transduced into cells by recombinant lentivirus to generate stable Rab18 knockdown cell lines (Figure 1a). As shown in Figure 1b, this approach yielded marked reduction of Rab18 both in gene and protein expression levels, with shRab18-3 showing the highest knockdown efficiency. Additionally, cell viability was not significantly affected by Rab18 knockdown (Figure 1c). Further, NTshRNA and shRab18-3 cell lines were infected with CSFV at an MOI of 1. After infection for 24, 48, and 72 h, cells and supernatants were collected for viral RNA quantification and viral titration, respectively. The results showed that, compared to those in NTshRNA cell lines, viral RNA copy numbers of CSFV and infectious virus production were significantly impaired in shRab18-3 cell lines (Figure 1d). To further validate that Rab18 silencing inhibited virus production, a rescue test was designed to determine whether the decrease in CSFV production could be due to off-target effects. Therefore, pEGFP-Rab18-mutants with silent mutations at the site targeted by shRab18-3 were obtained and verified by western blot (Figure 1e). As shown in Figure 1d, viral RNA copy numbers and virus titers were restored in shRab18-3 cells with transfection of pEGFP-Rab18-mutants. Taken together, these results revealed that Rab18 is required for CSFV infection.

### Rab18 expression positively regulates CSFV propagation

**Figure 2.**
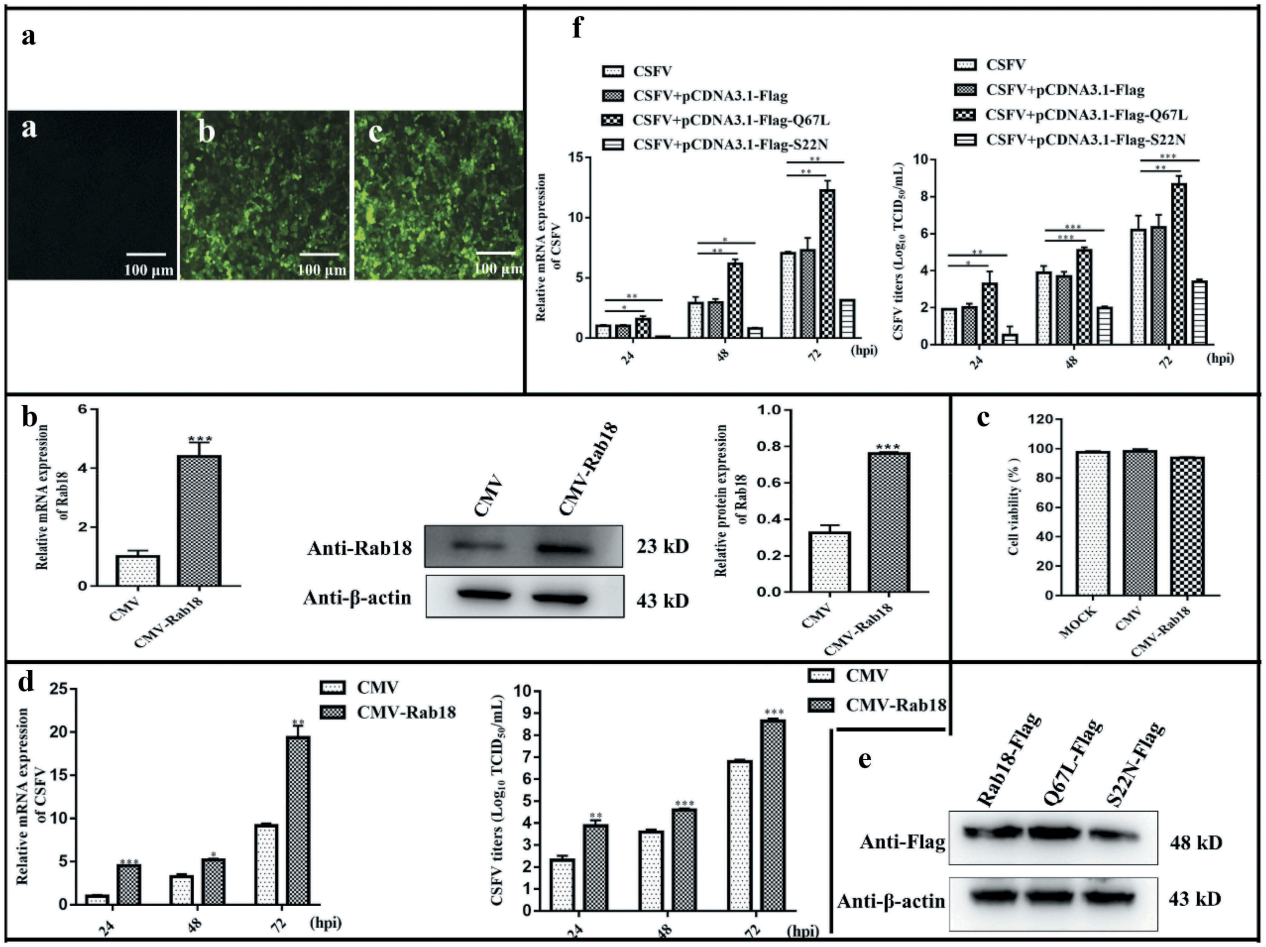
Overexpression of Rab18 enhances CSFV production. (a–c) Overexpression of Rab18 in SUVECs. (a) Confirmation of CMV and CMV-Rab18 cell lines by detection of the enhanced green fluorescent protein (EGFP) reporter. (**a**) Mock-transfected SUVECs. SUVECs transfected with lentiviruses expressing (b) CMV or (c) CMV-Rab18. Scale bars, 100 μm. (b) qRT-PCR and western blot analyzes Rab18 gene and protein expression levels in Rab18 overexpression cell lines. β-actin served as an internal control. (c) Cell viability of CMV-Rab18 cell lines. (d) qRT-PCR analyzes CSFV RNA level and TCID50 assay to detect CSFV viral titers in the supernatants of CMV cell lines and CMV-Rab18 cell lines. (e) Western blot analyzes pCDNA-Flag-Red-Rab18, pCDNA-Flag-Red-Rab18 (Q67L), and pCDNA-Flag-Red-Rab18 (S22 N) in transfected cells by anti-Flag antibody. β-actin served as an internal control. (f) qRT-PCR analyzes CSFV RNA level and TCID50 assay to detect CSFV viral titers in supernatants of pCDNA-Flag-Red-Rab18-, pCDNA-Flag-Red-Rab18(Q67L)-, and pCDNA-Flag-Red-Rab18(S22N)-transfected SUVECs. β-actin served as an internal control.

To further clarify the effects of Rab18 on CSFV proliferation, cell lines that stably overexpressed Rab18 (CMV-Rab18) and CMV were generated by recombinant lentiviruses (Figure 2a). The Rab18 gene and protein expression levels were determined by RT-qPCR and western blot, respectively (Figure 2b), and the cell viability was not significantly affected (Figure 2c). Further, the CMV and CMV-Rab18 cell lines were infected with CSFV (MOI = 1). Along with the increase in Rab18 expression, viral RNA copy numbers were significantly increased in CMV-Rab18 cell lines compared to CMV (Figure 2d). Moreover, the infectious particles in the supernatants of CMV-Rab18 cell lines were significantly increased (Figure 2d). Therefore, our findings indicated that overexpression of Rab18 promotes CSFV production.

Rab18, a member of the Ras superfamily of small GTPases, regulates membrane trafficking through cycling between inactive form Rab18-S22 N and active form Rab18-Q67L [22,33]. To further evaluate the involvement of Rab18 in CSFV infection, pcDNA-Flag-Red, pcDNA-Flag-Red-Rab18 (Q67L), and pcDNA-Flag- Rab18 (S22N) were transfected into cells, and the protein expression of transfected cells was detected by western blot with an anti-Flag antibody (Figure 2e). Subsequently, these cells were infected with CSFV (MOI = 1). As depicted in figure 2f, viral RNA copy numbers and infectious virus production were increased in cells with pcDNA-Flag-Red-Rab18 (Q67L) transfection. However, cells transfected with pcDNA-Flag-Red-Rab18 (S22N) showed opposite results. Collectively, these findings indicated that active form Rab18-Q67 L is required for CSFV infection.

### Rab18 is involved in replication and assembly steps of CSFV life cycle

**Figure 3.**
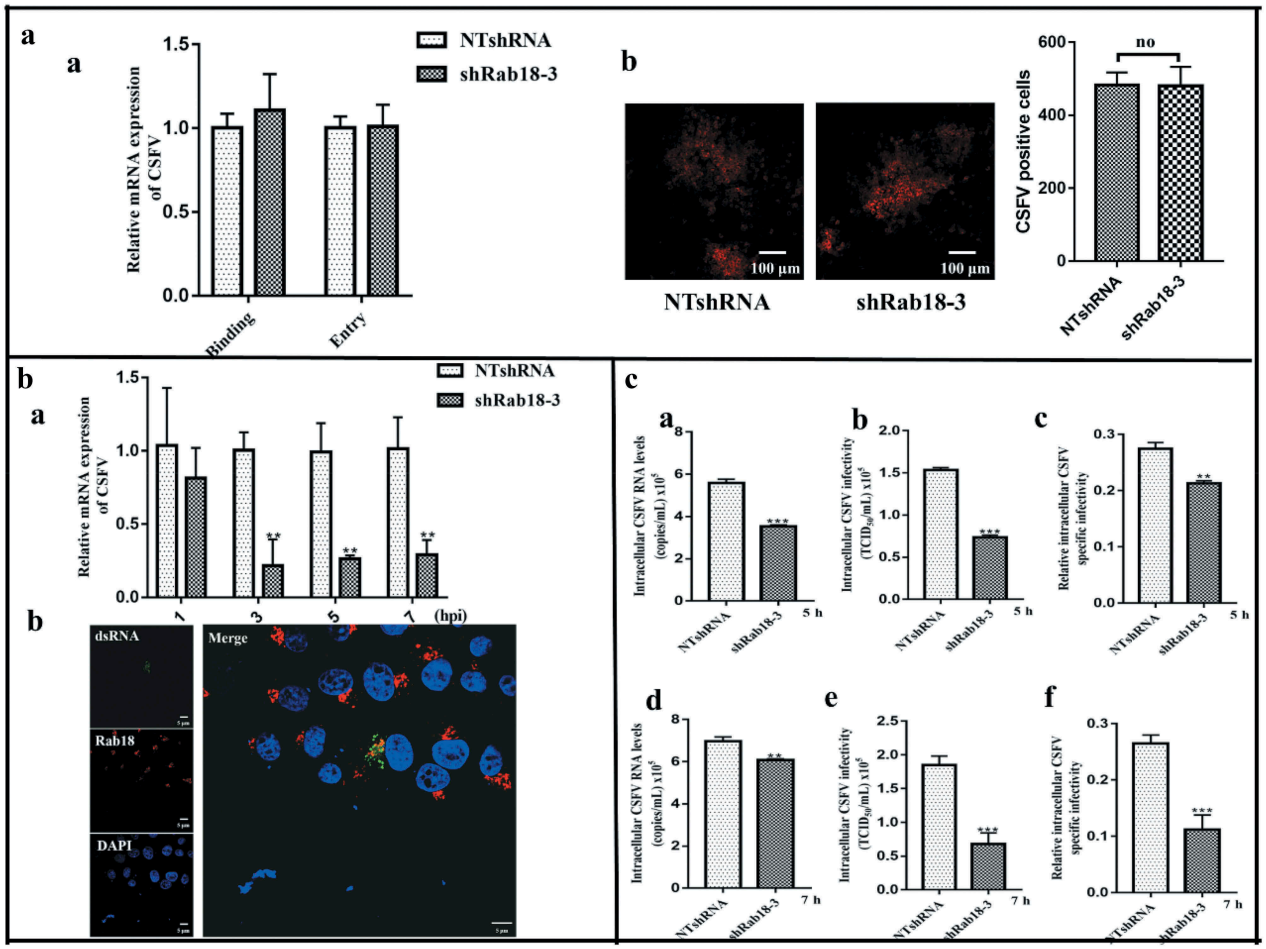
Rab18 is involved in CSFV replication and assembly steps. (a) (**a**) In the virus binding assay, NTshRNA and shRab18-3 cell lines were incubated with CSFV for 1 h at 4ºC, total cell RNA was extracted, and the CSFV RNA level was quantified by qRT-PCR. β-actin served as an internal control. In the virus entry assay, NTshRNA and shRab18-3 cell lines were incubated with CSFV for 1 h at 4ºC. Then, cells were washed with PBS and cultured in fresh medium at 37ºC for 2 h. Finally, total cell RNA was extracted, and the CSFV RNA level was quantified by qRT-PCR. β-actin served as an internal control. (b) Detection of CSFV entry by IFA. NTshRNA and shRab18-3 cell lines were infected with CSFV (5 MOI) for 1 h at 4ºC. Then, cells were washed with PBS and cultured in fresh medium at 37ºC for 2 h. Finally, cells were fixed with 4% paraformaldehyde, and immunofluorescence staining was performed using anti-E2 antibodies. Cells were counterstained with DAPI to label nuclei (blue). (b) Rab18 involvement in CSFV replication. (**a**) NTshRNA and shRab18-3 cell lines were incubated with CSFV at different time points (1, 3, 5, 7 h), total cell RNA was extracted, and CSFV RNA level was quantified by qRT-PCR. β-actin served as an internal control. (**b**) pcDNA-Flag-Red-Rab18-transfected cells were incubated with CSFV (0.5 MOI). At 6 h post-infection, cells were fixed with 4% paraformaldehyde, and immunofluorescence staining was performed using anti-dsRNA antibodies. Cells were counterstained with DAPI to label nuclei (blue). Scale bars, 5 μm. (c) Rab18 involvement in CSFV assembly. (**a**) NTshRNA and shRab18-3 cell lines were infected with CSFV. At 5 h post-infection, intracellular CSFV RNA levels were determined by qRT-PCR. (**b**) NTshRNA and shRab18-3 cell lines were infected with CSFV, and 5 h post-infection, cell supernatants were removed and disrupted by multiple cycles of freezing and thawing. The viral titer was determined by the TCID50 assay. (**c**) Relative intracellular specific infectivity was calculated as a ratio of the intracellular viral infectivity to the intracellular CSFV RNA. (d) NTshRNA and shRab18-3 cell lines were infected with CSFV, and 7 h post-infection, intracellular CSFV RNA levels were determined by qRT-PCR. (e) NTshRNA and shRab18-3 cell lines were infected with CSFV, and 7 h post-infection, cell supernatants were removed and disrupted by multiple cycles of freezing and thawing. The viral titer was determined by the TCID50 assay. (f) Relative intracellular specific infectivity was calculated as a ratio of the intracellular viral infectivity to the intracellular CSFV RNA.

Early studies have shown that Rab GTPases are involved in different proliferation steps of several viruses. Therefore, we explored the effect of Rab18 on the CSFV life cycle. We first investigated the functional role of Rab18 in CSFV binding and entry. For virus binding, NTshRNA and shRab18-3 cell lines were incubated with CSFV (MOI = 5) at 4°C for 1 h, and unbound virus was removed by washing with PBS. Following, cells were collected for RNA isolation to detect virus binding by RT-qPCR. For virus entry, NTshRNA and shRab18-3 cell lines were incubated with CSFV (MOI = 5) at 4°C for 1 h, unbound virus was removed by washing with PBS, and 2% medium was added to culture for an additional 2 h at 37°C. Finally, cells were collected for RNA extraction to detect virus entry by RT-qPCR. In addition, the effect of Rab18 on CSFV entry steps was detected by IFA. As shown in Figure 3a, we observed that CSFV binding and entry were not affected by Rab18 silencing, compared with those in NTshRNA cell lines. To determine whether Rab18 was involved in CSFV RNA replication, NTshRNA and shRab18-3 cell lines were harvested and RNA extracted at different time points (1, 3, 5, and 7 h). After CSFV (MOI = 5) infection, we observed that Rab18 silencing impaired viral RNA replication (Figure 3b). Given the effect of Rab18 on CSFV RNA replication, we observed co-localization of Rab18 and dsRNA in CSFV-infected cells (Figure 3b). We next examined whether Rab18 was involved in later steps of the CSFV life cycle. NTshRNA and shRab18-3 cell lines were incubated with CSFV (MOI = 5), and viral RNA copy numbers and titers of the intracellular virus were determined at 5 and 7 h post-infection. As shown in Figure 3c, viral RNA copy numbers and intracellular virus production were markedly decreased in shRab18-3 cell lines, indicating that CSFV assembly was Rab18 dependent. Taken together, these results showed that Rab18 is required for CSFV replication and assembly.

### CSFV NS5A binds to Rab18

**Figure 4.**
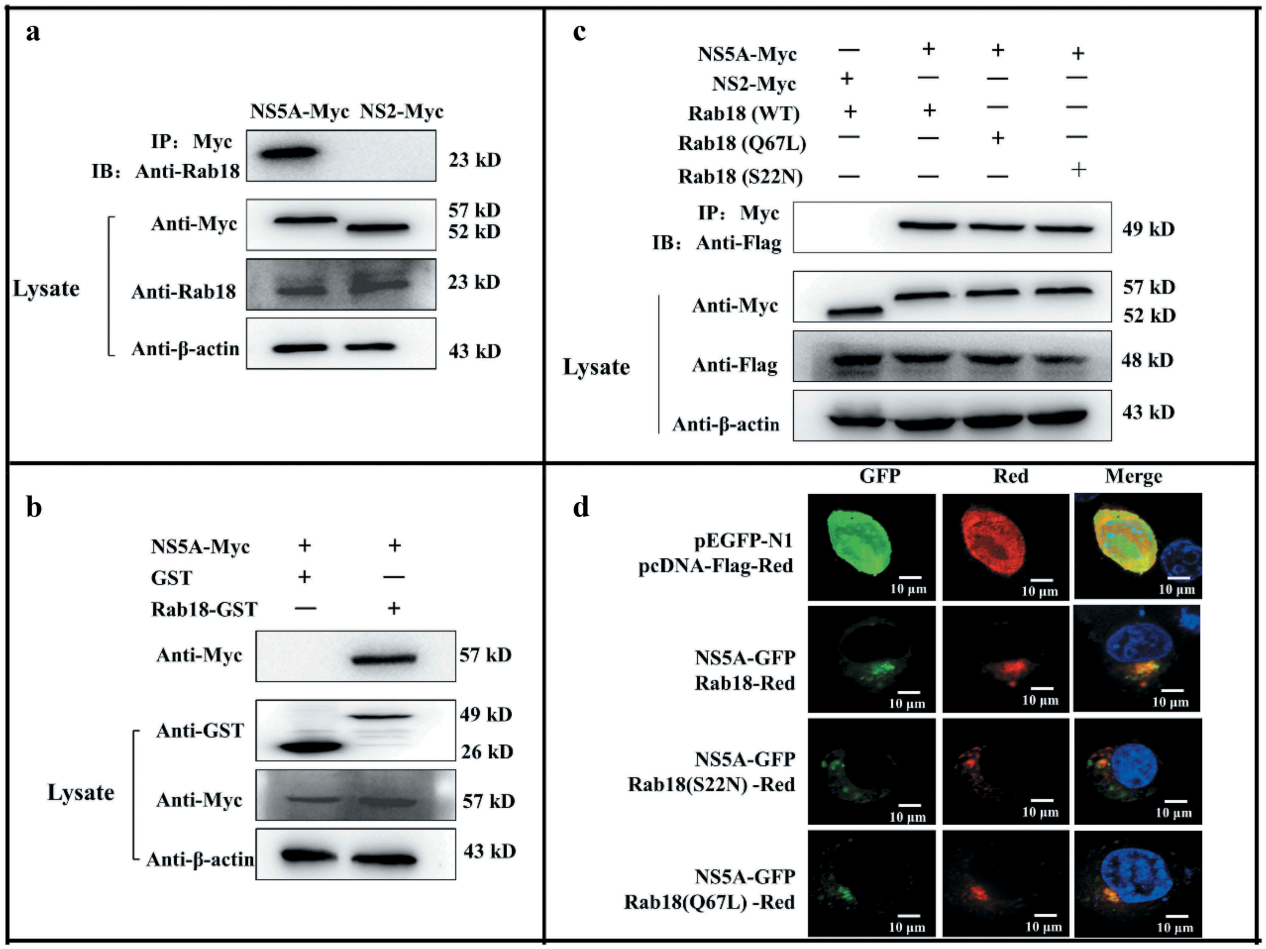
Rab18 binds to CSFV NS5A. (a) Co-immunoprecipitation (co-IP) assay. Cells were transfected with pcDNA-NS2-Myc or pcDNA-NS5A-Myc for 48 h. The transfected cells were lysed and immunoprecipitated, and western blot analysis was conducted using anti-Myc, anti-Rab18, and anti-β-actin. (b) GST-pulldown assay. GST or GST-Rab18 fusion proteins expressed in *E. coli* BL21 (DE3) were purified with glutathione agarose resin and incubated with the lysate of NS5A-Myc-expressing cells. Western blot analysis using anti-GST, anti-Myc, and anti-β-actin. (c) Exogenous NS5A-Myc binds to Rab18-Flag, S22N-Flag, and Q67 L-Flag in co-transfected cells. pcDNA-NS5A-Myc with pCDNA3.1-Rab18-Flag-, pCDNA3.1-S22N-Flag-, and pCDNA3.1-Q67L-Flag-transfected cells were lysed and immunoprecipitated. Then, western blot analysis was conducted using anti-Myc, anti-Flag, and anti-β-actin. pcDNA-NS2-Myc with pCDNA3.1-Rab18-Flag was used as a negative control. (d) Rab18 co-localization with CSFV NS5A protein. Cells were co-transfected with NS5A-GFP and Rab18-Red, NS5A-GFP and Q67L-Red, and NS5A-GFP and S22N-Red. Plasmids pEGFP-N1 and pCDNA-Red were co-transfected as a control. At 48 h after transfection, cells were fixed in 4% paraformaldehyde and stained with DAPI to label nuclei (blue). Scale bars, 10 μm.

Given the role of the non-structural protein NS5A in CSFV replication and assembly, anti-cMyc agarose affinity gel was used to immunoprecipitate the lysates from NS5A-Myc-transfected cells. Western blot analysis with the anti-Rab18 antibody showed that NS5A bound to endogenous Rab18 (Figure 4a). To further test whether the interaction between NS5A and Rab18 was direct, GST-pulldown assay was performed using GST-Rab18 protein expressed and purified from bacteria. As shown in Figure 4c, GST-fused Rab18, but not GST, could pull down NS5A. These findings collectively demonstrated that NS5A could directly interact with Rab18. To investigate whether NS5A selectively interacted with different forms of Rab18, cells were co-transfected with NS5A-Myc and either Rab18-Flag, Q67L-Flag, or S22N-Flag. Co-IP assay showed that NS5A bound to different forms of Rab18 (Figure 4c).

Since we previously demonstrated that Rab18 bound to NS5A, we determined whether Rab18 co-localized with NS5A in cells. Cells were co-transfected with NS5A-GFP and Rab18-Red, Rab18 (Q67L)-Red, or Rab18 (S22N)-Red. Confocal fluorescence microscopy revealed that CSFV NS5A co-localized with the wild type (WT) and both mutant forms of Rab18 (S22N and Q67L) (Figure 4d).

### Redistribution of NS5A in Rab18 knockdown cell lines

**Figure 5.**
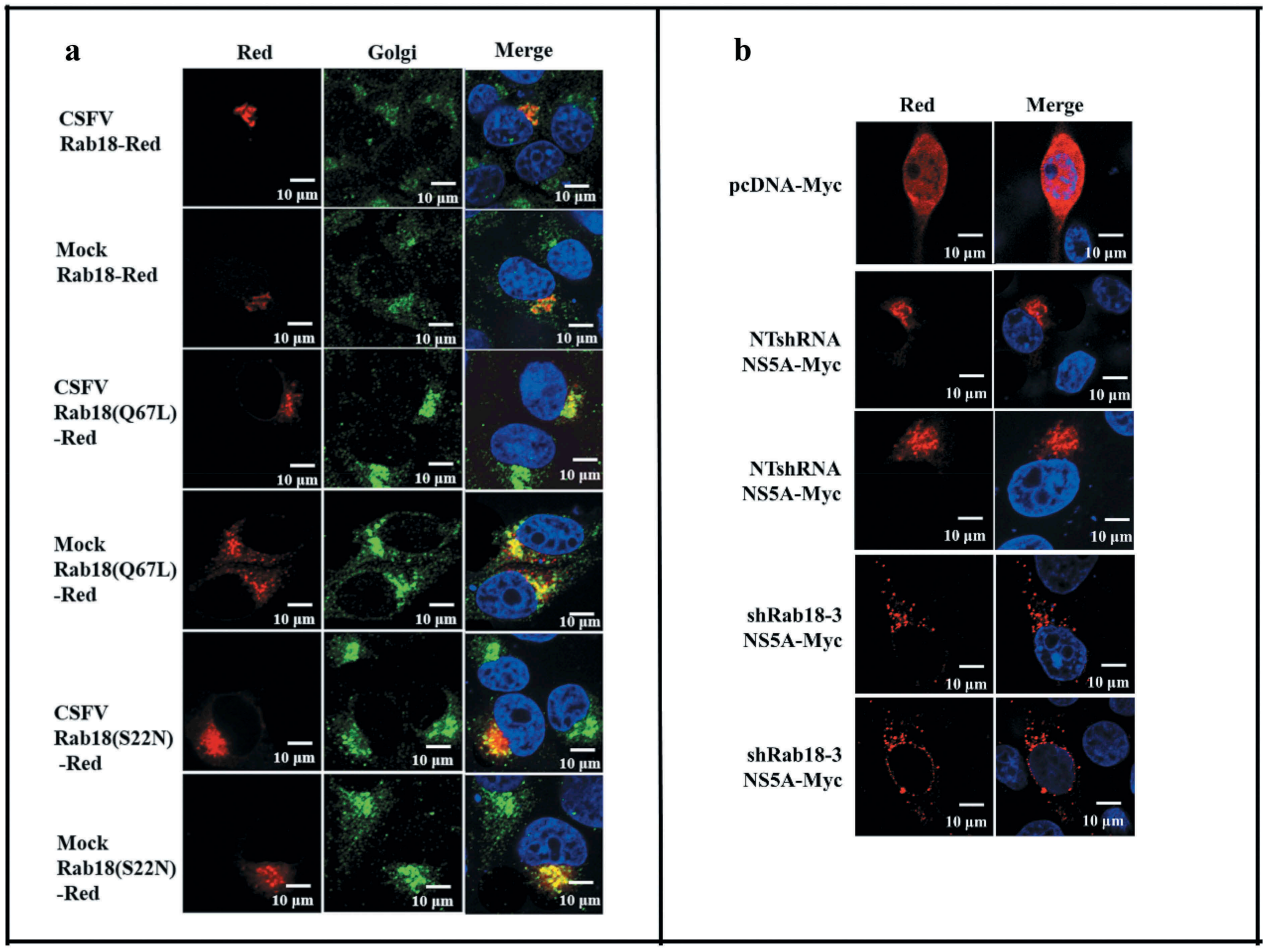
Redistribution of NS5A in Rab18 knockdown cells. (a) Rab18 localization at the Golgi apparatus. Cells seeded on glass coverslips were transfected with a Rab18-Red, Rab18 (Q67L)-Red, or Rab18 (S22N)-Red expression plasmid, as indicated. At 48 h after transfection, cells were fixed in 4% paraformaldehyde, and immunofluorescence staining was performed using an anti-TGN46 antibody. Cells were also counterstained with DAPI to label nuclei (blue). Scale bars, 10 μm. (b) NS5A distribution in a punctate pattern. NTshRNA and shRab18 cell lines seeded on glass coverslips were respectively transfected with pcDNA-Myc and NS5A-Myc expression plasmids, as indicated. At 48 h after transfection, cells were fixed in 4% paraformaldehyde, and immunofluorescence staining was performed using an anti-Myc antibody. Cells were also counterstained with DAPI to label nuclei (blue). Scale bars, 10 μm.

Following characterization of the role of Rab18 in CSFV production, we examined the subcellular distribution of WT and mutant forms of Rab18 protein in CSFV-infected cells. Rab18-Red, Rab18 (Q67L)-Red, and Rab18 (S22N)-Red expression plasmids were constructed and transfected into CSFV (1 MOI) infection cells. As shown in Figure 5a, different forms of Rab18 were located in the Golgi. Given the distribution of Rab18 in CSFV-infected cells and binding to NS5A, we speculated that Rab18 might regulate the distribution of NS5A in CSFV-infected cells. To substantiate this hypothesis, NTshRNA and shRab18-3 cell lines were transfected with NS5A-Myc, and confocal fluorescence microscopy analysis was conducted, which revealed that Rab18 knockdown led to punctate distribution of NS5A in CSFV-infected cells (Figure 5b). Together, these results suggested that Rab18 is a positive factor for CSFV production, probably by binding to the viral protein NS5A to support CSFV replication and assembly.

## Discussion

Viruses usually exploit the vesicle transport system to achieve invasion, replication, assembly, and secretion. Rab proteins, typically serving as molecular switches to mediate vesicular transport between membrane organelles, have been shown to be required for production of certain viruses [34]. Rab18 has been shown to be involved in the endocytic system, secretory pathway, and cell autophagy [22,35,36]. In a recent study, Rab18 was found to enhance HCV, BK polyomavirus, and DENV replication [26–29]. However, its functional involvement in CSFV infection has not been clearly defined. In this study, Rab18 knockdown and overexpression cell lines were generated by recombinant lentivirus to demonstrate the potential role of Rab18 in CSFV infection. Our results showed that CSFV replication was inhibited in Rab18-S22N-transfected cells and Rab18-silenced cell lines but enhanced in Rab18-Q67L-transfected cells and Rab18 overexpression cell lines, indicating the positive role of Rab18 in CSFV infection. Indeed, our previous studies have confirmed that Golgi-ER trafficking regulators of Rab1A and Rab2 were required for CSFV infection and suggested that trafficking of vesicles between the Golgi apparatus and the ER was important for CSFV production [17,32]. These results also suggested that vesicular transport mediated by Rab18 might play an important role in the CSFV life cycle. Therefore, besides Rab1A, Rab2, Rab5, Rab7, and Rab11, Rab18 is considered a novel Rab protein essential for CSFV infection.

In eukaryotic cells, viruses can rearrange in different intracellular membrane organelles to promote self-production [37]. Therefore, it is reasonable for viruses to utilize Rab proteins that are located in different membrane organelles for replication. During viral invasion, an enveloped virus enters Rab5-positive early endosomes and Rab7-positive late endosomes successively, and some viruses are then absorbed into Rab11-positive recycling endosomes [34]. Rab proteins are also required during the late period of viral replication, including virus assembly and release. Rab1, which regulates vesicular transport from the Golgi apparatus to the plasma membrane, is required for HCV release [38]. Rab7 activity upregulation in HBV-infected cells promoted virus release, probably by reducing viral trafficking to endosomal, lysosomal, and autophagic secretion systems [39]. Some viruses often utilize the same Rab proteins in different steps of their life cycle. For example, Rab7 was required for HIV particle maturation and secretion [40]. Rab18 is known as a regulator of endosomal transport and retrograde Golgi-to-ER transport in eukaryotic cells [35,41]. To further explore which step of CSFV infection is Rab18-dependent, we systematically dissected the role of Rab18 in the CSFV life cycle. Our results showed that Rab18 did not influence CSFV binding and entry in SUVECs, indicating that Rab18 was not essential during the endocytosis process of CSFV invasion in SUVECs. However, in previous studies, Rab18 has been shown to be involved in the endocytosis process [41], yet no direct evidence has shown that virus invasion is affected by Rab18. Like HCV and BVDV, other members of the *Flaviviridae* family, CSFV enters cells occurs through endosome-dependent invasion pathways [42,43]. CSFV enters PAM cells through caveolae-mediated endocytosis-early endosome (Rab5)-late endosome (Rab7)-recycling endosome pathways [44,45]. Moreover, CSFV entry into PK-15 cells occurs by clathrin-mediated endocytosis-early endosome (Rab5)-late endosome (Rab7) pathways [30]. Hence, further detailed studies are needed to elucidate the function of Rab18 in CSFV entry in other cell lines.

Based on our previous results, we chose four time points (1, 3, 5, and 7 h) to explore the role of Rab18 in CSFV RNA replication. Our results showed that CSFV genome copies were decreased in Rab18-silenced cell lines at different time points, compared with NTshRNA cell lines. Our results also showed co-localization of CSFV dsRNA and Rab18 in CSFV-infected cells by confocal microscopy. Consistent with previous studies, HCV RNA replication was inhibited in Rab18 knockdown cells, indicating the essential role of Rab18 in viral RNA replication [27]. These findings suggested that Rab18 might act as a host factor on the CSFV replication complex to enhance genome replication. Additionally, Rab27a is located in HCV RNA replication complexes to promote viral RNA replication in HCV-infected cells [46]. Intracellular detection of mature virus was not observed by TCID50 assay at 1 and 3 h post-infection. Therefore, viral titers of intracellular CSFV were quantified by TCID50 assay at 5 and 7 h post-infection, and the relative intracellular specific infectivity was calculated as a ratio of the intracellular viral infectivity to the intracellular CSFV RNA. Consistent with previous studies [26,27], we showed that the intracellular specific infectivity was decreased in shRab18 cells compared to NTshRNA cells, indicating that Rab18 was required for virion assembly.

Accumulating studies have indicated that the interactions between cell proteins and NS5A are essential to CSFV RNA replication and virion assembly [12–14]. Rab proteins serve as molecular switches, cycling between the inactive GDP-bound form and active GTP-bound form. GTP-bound-Rab proteins were mainly located in membranes, while GDP-bound-Rab proteins were located in the cytosol [47]. C-terminal cysteine residues of GDP-bound Rab proteins are prenylated by geranylgeranyl transferase [48]. After prenylation, Rab proteins are able to relocate to a special membrane, where GEFs (guanine-nucleotide exchange factors) interact with Rab proteins and exchange the bound GDP for GTP. Further, GTP-bound Rab proteins interact with effectors to mediate vesicular transport [19]. Once on the target membrane, GTP-bound Rab proteins are hydrolyzed by GTPase-activating proteins (GAPs) to GDP-bound Rab proteins, then GDP-bound Rab proteins are released from the membrane to the cytosol by GDP dissociation inhibitors (GDIs) [19,49]. Previous studies have confirmed that the interaction between Rab18 and NS5A can promote HCV assembly [27]. Further, our results revealed direct binding of NS5A and Rab18 by Co-IP and GST-pulldown assays, indicating that the interaction between NS5A and Rab18 may mediate the virus RNA replication and assembly. Because GTP-bound Rab proteins were located in the membranes, while GDP-bound Rab proteins were mostly located in the cytoplasm, we examined the interaction between NS5A and different forms of Rab18. Our results revealed that NS5A bound to the inactive form of GDP-Rab18(S22N) and active form GTP-Rab18(Q67L), indicating that Rab18 may serve as a carrier to regulate NS5A location in CSFV-infected cells.

Anterograde and retrograde trafficking of vesicles between the Golgi apparatus and the ER are regulated by Rab proteins [50], including Golgi-located protein Rab18 [22]. As shown in our study, Rab18, Rab18 (S22N), and Rab18 (Q67L) were mainly located in the Golgi apparatus in both cells and CSFV-infected cells, which is consistent with previous reports [22]. Considering that Rab18 has the function of regulating vesicle trafficking between Golgi and ER, and Rab18 was found to enhance HCV infection through trafficking of C and NS5A protein to assembly sites [26,27], we tested the distribution of NS5A in Rab18 knockdown cell lines. Our results showed that the distribution of NS5A was changed from aggregate distribution in NTshRNA cells to punctate distribution in Rab18-silenced cells, which was also observed in GDP-Rab18(S22N)-transfected cells, indicating that Rab18 can modulate the distribution of CSFV NS5A protein.

Because CSFV RNA copies and virion assembly were decreased in Rab18 knockdown cells, the role of Rab18 in virus secretion cannot be clearly defined. However, reduction of relative intracellular and extracellular specific infectivities in shRNA18 cells may indicate a role of Rab18 in virus secretion (data not shown). In conclusion, our results revealed Rab18 as a novel host factor, which is required for CSFV production and plays an essential role in the virus RNA replication and assembly steps of CSFV infection.
